# Clinical Use of Home Spirometry in Children With Cystic Fibrosis

**DOI:** 10.1002/ppul.71691

**Published:** 2026-06-09

**Authors:** Lucy Tan, Andrew Borowiec, James A. Reed, Mark Rath, Reid Masi, Laura Bennett, Don B. Sanders, Daniel J. Weiner, Clement L. Ren

**Affiliations:** ^1^ Division of Pulmonary and Sleep Medicine Children's Hospital of Philadelphia Philadelphia Pennsylvania USA; ^2^ Division of Pediatric Pulmonology, Allergy, and Sleep Medicine Riley Hospital for Children at Indiana University Health Indianapolis Indiana USA; ^3^ Department of Pediatric Pulmonology University of Pittsburgh Medical Center Children's Hospital of Pittsburgh Pittsburgh Pennsylvania USA; ^4^ Department of Biomedical and Health Informatics Children's Hospital of Philadelphia Philadelphia Pennsylvania USA

## Abstract

**Background:**

The use of home spirometry (HSPIR) has increased in pediatric cystic fibrosis (CF) care, but how it has been used clinically and its impact on clinical care have not been described. The purpose of this study was to address this knowledge gap through a secondary analysis of data from a HSPIR quality improvement project to characterize clinical use of HSPIR in children with CF (CwCF).

**Methods:**

HSPIR devices were distributed to 161 CwCF (age ≥ 5 years) across three CF centers. Remote encounters were reviewed to identify HSPIR use from July 2023 to February 2025. Data collected included the clinical indication for testing and its impact on clinical decision making. Differences between HSPIR users and non‐users were analyzed using Wilcoxon rank‐sum, Pearson's chi‐squared, and Fisher's exact tests as appropriate, and descriptive statistics were used to characterize clinical HSPIR use.

**Results:**

Among 161 CwCF who were provided HSPIR devices, 50 (31%) used their HSPIR a total of 107 times and submitted tracking forms to their care teams over 20 months. There were no significant differences in demographics or clinical characteristics between HSPIR users and non‐users. The most common reasons for HSPIR use were clinic follow‐ups (44%) and sick calls (33%), with clinicians reporting that HSPIR data informed clinical decisions in 72% of encounters. In 41% of remote clinical encounters, we identified a missed opportunity for using HSPIR.

**Conclusion:**

In patients and families motivated to use HSPIR, its use avoided the need to return to the clinic for follow‐up spirometry and influenced clinical decision making, suggesting that HSPIR can be a useful clinical tool. The overall low uptake of HSPIR highlights the need for further research to address patient and clinician barriers to its use. The impact of HSPIR on long‐term CF outcomes also requires further study.

## 1 Introduction

The COVID‐19 pandemic triggered a rapid and widespread adoption of telehealth care [1].

Spirometry, which is a crucial tool in lung disease assessment, was limited by the lack of in‐person visits at the time. As a result, there was a shift toward increasing use of home spirometry (HSPIR) as a remote monitoring tool for chronic respiratory conditions [2, 3]. Its use in cystic fibrosis (CF) has been especially important because of the condition's need for frequent lung function monitoring [4]. Participants who used HSPIR have shared positive feedback regarding convenience and expressed interest in continuing its use [5, 6].

While HSPIR has potential benefits, its use in children with CF (CwCF) presents distinct challenges, prompting most studies in this area to focus on assessing its reliability and accuracy in this population. Adults with CF have been able to produce consistent spirometry results on home devices [7] and effectively track longitudinal changes in lung function [8], whereas measurements in children are more variable [9, 10]. However, with structured coaching and training, children and adolescents have been shown to produce reliable HSPIR results [11, 12].

Furthermore, studies performed in Turkey and France have shown that HSPIR may play a role in the early detection of pulmonary exacerbations [13, 14]. CF clinicians are limited in their use and interpretation of HSPIR due to multiple factors, including the lack of standardized HSPIR protocol [15], its variable feasibility [5], and lack of integration with the electronic health record.

Despite the growing literature describing the clinical use of HSPIR in CwCF, there remains a knowledge gap regarding how data collected from HSPIR can effectively support clinical decision‐making in routine practice. To address this gap, we performed a secondary analysis of data from an ongoing quality improvement (QI) initiative designed to increase the use of HSPIR amongst CwCF. While the data is drawn from a QI initiative, this study focused on the use of HSPIR in real‐world clinical settings and its potential clinical utility. Specifically, we aim to describe how it was used in clinical care and to determine its impact on clinical decision‐making. We hypothesized that we could identify specific clinical situations where HSPIR was used and that clinicians would use HSPIR information in clinical decision‐making.

## 2 Methods

Data from an ongoing QI project were used for this analysis. Implementing Clinical Use of Pediatric home Spirometry (ICUPS) is a multicenter QI project whose goal is to improve and increase the clinical use of HSPIR in CwCF. HSPIR devices were distributed at three CF centers: Children's Hospital of Philadelphia (CHOP), Riley Hospital for Children, and Children's Hospital of Pittsburgh (CHP). The project was reviewed by the Institutional Review Boards of the Children's Hospital of Philadelphia, Indiana University Health, and the University of Pittsburgh. Each determined that the project qualified as QI and granted a waiver of formal IRB approval and informed consent. All CwCF aged ≥ 5 years were considered candidates for HSPIR. Each CF Center made its own decisions about whom to give HSPIR to, and factors such as adherence to clinic visits and treatments, ability to perform clinic spirometry, and a reliable means of communication were considered in dispensing HSPIR.

Demographic data including age, sex, race, ethnicity, and CF‐specific medical history were collected. The Mir Smart One HSPIR (MIR‐Medical International Research, New Berlin, WI) was provided to a total of 161 CwCF (65 at CHOP, 46 at CHP, and 50 at Riley). HSPIR devices were distributed between 2023 and 2024. At the time of device distribution, each participant received brief in‐clinic instructions limited to demonstrating basic device operation. No standardized protocol or additional supervised practice was provided, and the content and delivery of training varied across sites. At CHOP and CHP, physical and respiratory therapists provided a single in‐clinic training session that included mobile application installation, device connectivity, and spirometer use. Links to instructional videos on YouTube were provided, though their utilization was not tracked. At CHP, written instructions were provided in clinics or mailed along with spirometers in some cases. At Riley, a similar approach was used without video resources, with an added emphasis on correlating home and clinic spirometry to ensure repeatability and build patient confidence. Additional education was provided at follow‐up visits at all three sites as needed. A baseline forced expiratory volume in 1 s (FEV_1_) was measured using the devices while participants were clinically stable. After setup, participants received no explicit or standardized directions on when to use the device, relying instead on CF team communication as needed. No remote or virtual coaching was offered during home testing, and participants used only the instructions included within the app. The results were then accessed by the child's primary CF provider using an online dashboard (ZephyRx, Troy, NY).

The use of HSPIR devices was tracked from July 2023 to February 2025. Tracking forms were recorded by the children's CF care teams with each reported use (see Supporting Information for sample tracking form). The recorded data included the FEV_1_, the type of associated clinical encounter, and whether the data contributed to clinical decision‐making. Tracking forms were completed in response to remote communications, such as phone calls or portal messages, particularly when new symptoms arose, and sometimes during scheduled follow‐up to monitor symptom progression or lung function. Encounters were defined as any remote clinical interaction of which the CF care team was aware, including remote communications such as phone calls or messages. Instances in which patients independently used the home spirometer without communicating with the care team were not considered encounters. Nurses at CHOP and CHP who handled calls or messages related to HSPIR use did not interpret spirometry results but instead notified CF providers, who were responsible for all clinical interpretation and management decisions. At Riley Hospital, respiratory therapists monitored the online dashboard, and the patient's CF provider was notified whenever a test was completed, either independently or during sick calls or exacerbation treatment. The CF providers reviewed the results in the context of these encounters, considering the quality of measurements, and subjectively determined whether the HSPIR results informed symptom assessment, follow‐up planning, or adjustments to care.

Clinical characteristics between HSPIR users and non‐users were compared using Wilcoxon rank‐sum, Pearson's chi‐squared, and Fisher's exact tests as appropriate. Data from the tracking forms were analyzed using descriptive statistics, including counts and percentages, to summarize the types of encounters during which HSPIR was performed. The impact on clinical decision‐making was assessed subjectively by providers, and the percentage of encounters in which providers indicated that the HSPIR results contributed to clinical decision‐making was calculated. The quality of the FEV_1_ measurements, according to American Thoracic Society (ATS) grading criteria, was summarized using counts.

We also tracked the number of missed opportunities, that is, remote clinical encounters where HSPIR could have been used but was not. The proportion of missed opportunities relative to HSPIR utilization was calculated by dividing the number of missed opportunities by the total number of remote clinical encounters, defined as patient portal messages and telephone calls in which HSPIR could have been utilized.

## 3 Results

All eligible patients approached for enrollment accepted HSPIR devices. Overall, 37% of eligible CwCF aged ≥ 5 years across the three sites received HSPIR devices: CHOP (34%), CHP (87%), and Riley (27%). HSPIR users were defined as those who submitted at least one tracking form documenting their use. The demographic and clinical features of HSPIR users compared with non‐users are described in Table 1. There were no significant differences between users and non‐users.

**Table 1:** Demographic and clinical features of HSPIR users and non‐users. Unless otherwise noted, all values are reported as number (%).

Characteristic	User (*N* = 50)^a^	Non‐user (*N* = 111)^a^	*p* value^b^
Age, mean (range)	12 (9, 15)	13 (10, 15)	0.13
Sex			0.7
Male	27 (54%)	56 (50%)	
Female	23 (46%)	55 (50%)	
Race			0.7
American Indian/Alaska Native	0 (0%)	0 (0%)	
Asian	0 (0%)	0 (0%)	
Black or African American	1 (2.0%)	2 (1.8%)	
Native Hawaiian or Other Pacific Islander	0 (0%)	0 (0%)	
White	47 (94%)	107 (96%)	
Other^c^	2 (4.0%)	2 (1.8%)	
Ethnicity			0.5
Hispanic or Latino	1 (2.0%)	1 (0.9%)	
Not Hispanic or Latino	49 (98%)	110 (99%)	
Baseline in‐clinic FEV1% predicted, mean (range)	104 (96, 114)	106 (94, 116)	0.6
Genotype			0.6
F508 homozygous	23 (46%)	58 (52%)	
F508 heterozygous	23 (46%)	41 (37%)	
Other	4 (8.0%)	12 (11%)	
CFTR modulator			0.8
Elexacaftor/tezacaftor/ivacaftor	44 (88%)	98 (88%)	
Ivacaftor	2 (4.0%)	3 (2.7%)	
Tezacaftor/ivacaftor	0 (0%)	2 (1.8%)	
Lumacaftor/ivacaftor	0 (0%)	2 (1.8%)	
None	4 (8.0%)	6 (5.4%)	
CF‐related diabetes	3 (6.0%)	24 (22%)	0.014
CF liver disease	6 (12%)	16 (14%)	0.7
Gastrostomy tube placement	8 (16%)	14 (13%)	0.6
Medications			
Use of Dornase alfa	45 (90%)	84 (76%)	0.035
Use of hypertonic saline nebulization	42 (84%)	83 (75%)	0.2
Use of azithromycin	14 (28%)	33 (30%)	0.8
Microbiology history^d^			
*Pseudomonas aeruginosa*	8 (16%)	21 (19%)	0.7
Methicillin‐resistant *Staphylococcus aureus*	6 (12%)	21 (19%)	0.3
Methicillin‐sensitive *Staphylococcus aureus*	40 (80%)	79 (71%)	0.2

^a^ Median (Q1, Q3); *n* (%).

^b^ Wilcoxon rank sum test; Pearson's Chi‐squared test; Fisher's exact test.

^c^ Includes individuals whose specific racial identity was not specified.

^d^ Culture history reflects any growth of these organisms in the patient's respiratory cultures within the past 2 years.

**Figure 1:**
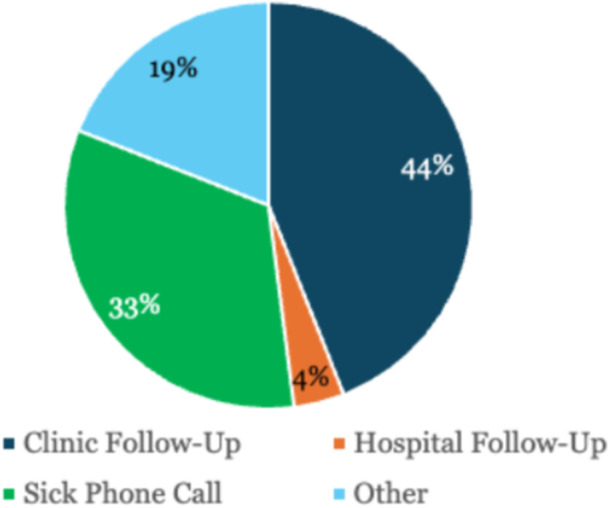
Types of clinical encounters for home spirometry use. Distribution of clinical encounter types during which HSPIR data were used (*n* = 107). Each segment represents the proportion of HSPIR tracking forms associated with a specific encounter type, including clinic follow‐ups, sick phone calls, post‐hospitalization follow‐ups, and other types. [Color figure can be viewed at wileyonlinelibrary.com]

From July 2023 to February 2025, there were 181 remote encounters, and 50 of the 161 patients with HSPIR devices (31%) used their devices a total of 107 times. There were 74 encounters (41% of the total) where HSPIR could have been used but was not, either because the patient did not use it or the care team did not request its use. The types of clinical encounters associated with each use are illustrated in Figure 1. Most clinical encounters where HSPIR was utilized were for clinic follow‐ups (44%) and sick phone calls (33%). Children were not asked to come to the CF center specifically for additional testing related to HSPIR use. Clinic follow‐ups were generally virtual check‐ins conducted after recent clinic visits to address concerns such as a decline in FEV_1_ percent predicted or symptoms of a pulmonary exacerbation requiring monitoring or treatment. The most common reason in the “Other” category was for pre‐clinic visit assessments. These HSPIR uses were often self‐initiated by patients with concerns and performed prior to a scheduled clinic visit, allowing clinicians to review HSPIR data and tailor care based on current trends or issues. Other reasons in this category included lung function monitoring following initiation of a new medication and virtual check‐ins for missed clinic visits.

**Figure 2:**
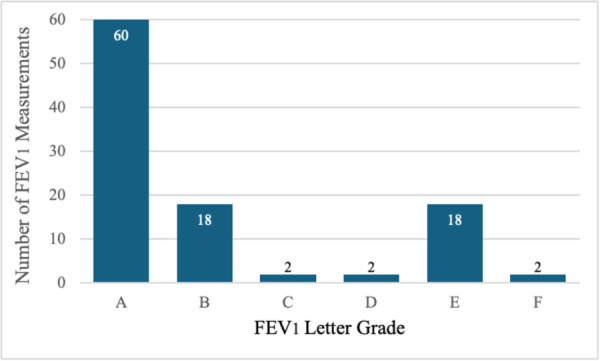
Distribution of ATS quality grades for FEV1 measurements submitted with tracking forms. Grades A and B indicate 2–3 acceptable measurements with repeatability within 150 mL. Grades C and D reflect acceptable measurements with lower repeatability. Grade E represents ≥ 2 acceptable measurements but with wide variability. Grade F indicates unusable measurements due to poor quality or lack of acceptable maneuvers. [Color figure can be viewed at wileyonlinelibrary.com]

In most encounters (72%), CF clinicians reported using HSPIR data to guide clinical decisions. Common reasons reported for not using the data include poor quality or technique and delays in clinician review. Among 107 tracking forms collected, 102 were available for review of effort and ATS spirometry quality grading of FEV_1_. Patients made a median of 4 (IQR 3–6; mean 5.22; range 1–21) spirometry attempts per HSPIR use before providing final results to their CF clinician. The distribution of ATS quality grades for FEV_1_ measurements is shown in Figure 2, with most tests achieving grade A (59%) or B (18%). There was no significant relationship between patient age and quality of the HSPIR measurements.

## 4 Discussion

While quality control and feasibility of HSPIR have been extensively studied, there are limited data on its role in routine clinical care and decision‐making. In this analysis of real‐world utilization of HSPIR in pediatric CF care, we found that clinic follow and sick phone calls were the most common reasons for HSPIR, and clinicians felt it provided useful clinical information in that large majority of cases. These results suggest that HSPIR can be a useful clinical tool, and our findings will be helpful for CF Centers considering how to implement HSPIR into their clinical care pathways.

Although only 31% of patients who received HSPIR devices used them and completed associated tracking forms, this subgroup participated in multiple clinical encounters using HSPIR, and most of their measurements contributed to clinical decision‐making. These findings support the implementation of HSPIR into clinical care amongst an engaged subset of CwCF, but they also highlight challenges to broader implementation, as uptake was limited and integrating HSPIR into routine care remains complex.

HSPIR influenced clinical decision‐making in the majority (72%) of encounters, reflecting both its potential and limitations in practice. In the remaining 28% of cases, clinicians reported reasons for not using HSPIR data including data quality, patient technique, and lack of timely clinician review. Although most FEV_1_ measurements met ATS criteria for acceptable quality, performance may have been affected in some instances when patients were actively symptomatic, thereby potentially compromising HSPIR data reliability. Additionally, clinicians may not have reviewed HSPIR data in some cases because clinical decisions were already made based solely on symptoms, for example, a prolonged, wet cough. Therefore, HSPIR may be most useful as a complementary resource alongside clinical evaluation.

Our results further underscore the role of HSPIR in the evaluation and management of pulmonary exacerbations, particularly in the context of sick phone calls. CF clinicians used HSPIR results to guide treatment decisions, including whether to start or adjust antibiotics, the duration of therapy, and modifications to other treatments such as airway clearance routines. These results also helped determine whether an in‐person evaluation or additional tests were necessary, or if the patient could safely continue care at home. This practice corresponds to increased HSPIR use by CF patients during periods with increased pulmonary symptoms, as reported by Bertram et al. [15]. Prior studies have shown that HSPIR may play a role in early detection of pulmonary exacerbations [13], although the impact of treatment on long‐term lung function remains unclear due to conflicting findings [14, 16]. This study is, to our knowledge, the first to explore how clinicians incorporate HSPIR data into real‐time clinical decision‐making for patients with CF. By systematically examining clinician interpretation and use of HSPIR data across multiple institutions, our findings offer important insight to guide its integration into routine pediatric CF care.

A major limitation in our study is the low uptake of HSPIR with only 31% of those with devices using them, raising questions about degree to which HSPIR can be incorporated into clinical care. We did not observe differences in demographics and clinical characteristics between HSPIR users and non‐users, although we were unable to assess other factors, such as adherence and motivation that could have affected HSPIR use. Prior studies have reported that adherence to HSPIR use generally declined over time, even among initially motivated patients [5, 17]. Overall, no technical issues, such as device malfunction or data transmission errors, were reported among HSPIR users. However, non‐users may have experienced barriers similar to those reported by Liu et al. [18], including technical difficulties, communication breakdowns, and a lack of specific feedback from the care team, all of which may have contributed to limited engagement with HSPIR. Patient perspectives were not included in the scope of our analysis, but future studies could explore these views to better understand factors influencing HSPIR use.

Several additional limitations should be considered. Enrollment at all three sites was influenced by prior in‐clinic spirometry performance and the clinical team's assessment of each child's ability to use the devices, which may have led to the exclusion of children who were less able or less motivated to complete home testing. The overall high quality of HSPIR, along with the lack of an observed association between age and quality, likely reflects underlying selection bias in the sampled population. No virtual coaching, aside from the in‐app instructions, was provided during remote testing, which may have affected quality and consistency of HSPIR results. However, this approach reflects real‐world use, where patients often complete HSPIR without direct supervision [19]. The impact of HSPIR results on clinical decision‐making was assessed subjectively by clinicians, and we did not collect structured data on how these determinations were made. Additionally, reasons for not using HSPIR were only captured when voluntarily recorded on the tracking form, which may have introduced reporting bias and limited our understanding of factors influencing its use.

In summary, our study has described how HSPIR is used clinically at three CF Center in the USA, and that HSPIR provides clinically useful information in an engaged subset of CwCF. Further work is needed to identify and mitigate barriers to great use of HSPIR and to assess its impact on long‐term outcomes.

## Author Contributions

**Lucy Tan:** writing – original draft, data curation, investigation, formal analysis, visualization, funding acquisition. **Andrew Borowiec:** data curation, project administration, investigation. **James A. Reed:** investigation. **Mark Rath:** investigation. **Reid Masi:** investigation. **Laura Bennett:** formal analysis. **Don B. Sanders:** writing – review and editing. **Daniel J. Weiner:** writing – review and editing. **Clement L. Ren:** conceptualization, formal analysis, methodology, supervision, writing – review and editing, and funding acquisition.

## Conflicts of Interest

The authors declare no conflicts of interest.

## Supporting information

Supporting File

## Data Availability

The data that support the findings of this study are available on request from the corresponding author. The data are not publicly available due to privacy or ethical restrictions.
