# Supplementary material for: Clinical Use of Home Spirometry in Children With Cystic Fibrosis
Source: Pediatr Pulmonol. 2026 Jun 9;61(6):e71691. doi: 10.1002/ppul.71691 (PMC13247608; doi:10.1002/ppul.71691)
Supplement: Supplementary file 1 — Supporting File [file PPUL-61-0-s001.pdf]

Home Spiro Tracking Form

Date of encounter:

Patient age (years):

Type of encounter :

Was home spirometer used :

Home spiro baseline FEV1 (% predicted):

Home spiro FEV1 at the encounter (% predicted):

Did clinician review the home spirometry data :

Effort and reproducibility acceptable (yes/no):

Was home spirometry data use in making a clinical decision (yes/no):

Other comments (free text):

Tracking forms completed by pediatric CF care teams to document each reported HSPIR use.
